# Association Between Oral Function and Underweight in Dental Outpatients Under Regular Maintenance: A Cross-Sectional Study

**DOI:** 10.7759/cureus.103897

**Published:** 2026-02-19

**Authors:** Ayaka Kayano, Tomomi Ohmaru, Hiroko Tsuda, Naohisa Wada

**Affiliations:** 1 Department of General Dentistry, Division of Interdisciplinary Dentistry, Faculty of Dental Science, Kyushu University, Fukuoka, JPN; 2 Department of General Dentistry, Kyushu University Hospital, Fukuoka, JPN

**Keywords:** bmi, cross-sectional study, dental maintenance, dental outpatients, oral hypofunction, underweight

## Abstract

Objective

This study aimed to evaluate the prevalence of oral hypofunction among dental maintenance outpatients aged over 50 years who had completed dental treatment at least three months before and to explore the relationship among oral hypofunction, age, and underweight.

Background

Recently, the relationship between oral function and general health, such as frailty or sarcopenia, through the mechanism of declining nutritional status, has been the focus of research. Improving oral function, such as general dental treatment, may prevent deterioration or improve overall health, but the causal relationship has not yet been fully clarified. It is useful how often the general dentist might encounter subjects with oral hypofunction in regular maintenance patients or how oral hypofunction could predict present underweight status.

Materials and methods

This cross-sectional study enrolled 107 subjects (mean age: 72.9 ± 9.2 years, 40.2% men). Oral hypofunction tests and body mass index (BMI) were used as indicators of oral function and nutrition status, respectively. Underweight was defined based on the BMI cut-off values specified in the Global Leadership Initiative on Malnutrition (GLIM) criteria for Asians. Other patient characteristics, such as medical history, the number of teeth, and functional tooth units (FTUs), were collected from the patients’ charts. The participants were classified into three groups: middle-aged (50-64 years), early older adult (65-74 years), and late older adult (≥75 years). Associations among oral hypofunction, underweight, and age were evaluated using two-way ANOVA, with additional analyses performed using general linear models (GLM) to account for potential confounders.

Results

The overall prevalence of oral hypofunction was 50.5%, and 48.1% of the participants in the nonunderweight group (n = 81) exhibited oral hypofunction. The oral hypofunction group was significantly older than the nonoral hypofunction group (75.5 ± 7.4 versus 70.3 ± 10.1 years, p < 0.05). No significant differences in BMI status were observed among the three age groups, although functions of oral hygiene status (tongue coating index {TCI}) and oral diadochokinesis (ODK) (/ta/ and /ka/) had significant differences in some parts of the age groups. In multivariable-adjusted general linear models, age group remained significantly associated with oral diadochokinesis, and a significant interaction between age group and underweight status was observed for tongue coating index.

Conclusion

Approximately half of the dental outpatients in the maintenance phase in this study exhibited oral hypofunction. Oral hypofunction was significantly observed in elderly people in the dental maintenance phase. Although it could not be detected as a significant uniform association between oral hypofunction and underweight, the small underweight group limits our ability to rule out a clinically meaningful relationship.

## Introduction

Recently, the relationship between oral function and general health, such as frailty or sarcopenia, through the mechanism of falling nutritional status, has been the focus of research [1]. Therefore, research has begun to hypothesize that improving oral function may prevent deterioration or improve overall health. However, other factors such as age and oral function are considered to influence general health, and the causal relationship has not yet been fully clarified [2,3].

In 2016, the Japanese Society of Gerodontology proposed the concept of “oral hypofunction,” which is diagnosed based on the evaluation result of seven oral function-related items [1]. Many studies have explored the true relationship between oral function and general disease or functions using this concept [4].

Among the many types of indicators to evaluate nutrition status, body mass index (BMI) is one of the most widely used simple indicators [5]. Underweight is associated with malnutrition risk or the need for long-term nursing care [6,7]. Clarifying which types of oral hypofunction are more likely to lead to nutritional deterioration or which patient characteristics, such as aging, are more closely related to malnutrition for predicting high-risk patients results in meaningful information. Moreover, this approach could lead to improved efficiency of medical resource allocation.

However, how dental diseases such as caries and periodontitis or tooth loss will decline the oral function, which severity and/or period of illness is more affected by the declining oral function, how conventional dental treatment can restore the loosened oral function, and whether additional treatment or training is needed to recover their function are unclear.

As for the first question, information on how often the general dentist encounters subjects with oral hypofunction in regular maintenance patients or how oral hypofunction could predict present underweight status may be useful.

In this study, the primary outcome was the prevalence of oral hypofunction, assessed based on the seven-item criteria of the Japanese Society of Gerodontology, and the primary exposure was underweight, defined according to the BMI cut-off values specified in the Global Leadership Initiative on Malnutrition (GLIM) criteria for Asians.

This study aimed to (1) evaluate the prevalence and characteristics of oral hypofunction among dental outpatients in regular maintenance aged over 50 years and (2) explore the relationship among oral hypofunction, age, and underweight as an indicator of nutritional status in this population.

We hypothesized that residual oral hypofunction would be correlated with malnutrition, independent of age, even in patients in the maintenance phase of dental treatment, and used a cross-sectional design to test this hypothesis. The null hypothesis was that no significant association exists between oral hypofunction and underweight.

## Materials and methods

**Figure 1:**
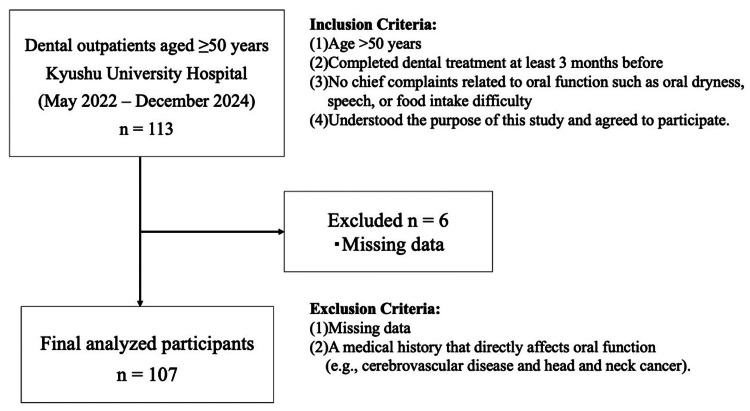
Flowchart of patient recruitment and selection

Subjects recruited were outpatients who visited the Department of General Dentistry, Kyushu University Hospital, between May 2022 and December 2024. This department offered initial training for new graduate dentists. Patients who did not have serious, complex medical backgrounds or require highly specialized dental treatment were accepted and provided relatively simple and fundamental dental treatments. The inclusion criteria were as follows: (1) age of >50 years; (2) completed dental treatment at least three months before; (3) no chief complaints related to oral function such as oral dryness, speech, or food intake difficulty; and (4) understood the purpose of this study and agreed to participate. No exclusion criteria were applied regarding the type of general dental treatment received (e.g., caries, periodontal disease, and prosthetics such as crowns or dentures). The exclusion criteria included those with missing data and a medical history that directly affects oral function (e.g., cerebrovascular disease and head and neck cancer) (Figure 1). The participants with missing data were excluded using a complete-case analysis approach. Missing data were primarily due to unanswered items in the questionnaire. No substantial differences in basic characteristics were observed between the participants included in the analysis and those who were excluded. Among outpatients who visited the Department of General Dentistry, Kyushu University Hospital, during the study period, the patients who met our inclusion criteria were recruited as subjects serially. Consent to participate in the study was obtained from those who accepted our offer. The participants were classified into three groups: middle-aged (50-64 years), early older adults (65-74 years), and late older adults (≥75 years) [8]. These thresholds align with commonly accepted gerontological thresholds that reflect distinct stages of aging and are frequently used in studies of oral function and frailty. The Kyushu University Hospital Institutional Review Board approved the study protocol (approval number: 2023-34), and written informed consent was obtained from all the participants.

Participant characteristics

Age, sex, medical history, the number of present teeth, and functional tooth units (FTUs) were obtained from dental records or interviews. BMI was calculated as weight (kg) divided by height squared (m²). According to the GLIM criteria, a low BMI in Asians is defined as <18.5 kg/m² for those under 70 years of age and <20.0 kg/m² for those over 70 years of age [5]. The subjects were classified into the nonunderweight and underweight groups using this cut-off value. FTUs (FTUs composed of natural teeth, fixed prostheses, and implants {nif-FTUs}) were defined as follows according to previous studies: FTUs are an index that evaluates the occlusal status on a scale of 0-12 points, with the occlusion of opposing premolars as one point and the occlusion of opposing molars as two points, and moreover, the nif-FTUs evaluate the occlusion of the natural teeth and fixed prostheses such as implants and bridges [9]. In this study, nif-FTUs were used for analysis.

Oral function tests

Oral function was evaluated based on the following seven-item criteria established by the Japanese Society of Gerodontology in 2016: oral hygiene status, oral dryness, occlusal force, oral diadochokinesis (ODK), tongue pressure, masticatory performance, and swallowing function (SF) [1]. The participants were diagnosed with “oral hypofunction” if abnormalities were observed in three or more of the seven items. For the patients included in this study, all measurements were performed during the approximately 10 minutes before the start of their routine dental maintenance procedures. All participants with dentures were examined while wearing their dentures.

All oral function assessments were performed by the authors (AK and TO), who were an experienced dental hygienist and dentist. Prior to data collection, both examiners underwent joint calibration and standardized their measurement procedures according to the protocols of the Japanese Society of Gerodontology (2016). Consistency in measurement procedures and the application of cut-off criteria was ensured before the main study. Inter-examiner reliability was investigated using intraclass correlation coefficients (ICCs). ICC values of >0.75 were considered indicative of excellent stability [10]. Examiner calibration focused on oral function measures with high examiner dependency, including tongue pressure, maximum occlusal force (MOF), and tongue coating index (TCI). The ICCs for the inter-examiner reliability values ranged from 0.849 (95% confidence interval {CI}: 0.629-0.944) to 0.983 (95% CI: 0.946-0.994). Other measurements were performed using standardized devices and protocols, minimizing examiner-related variability.

The criteria for each item were as follows: tongue coating index, oral moisture (OM), maximum occlusal force, tongue-lip motor function (oral diadochokinesis {ODK}), maximum tongue pressure (MTP), masticatory function (MF), and swallowing function.

Tongue Coating Index (TCI)

Oral hygiene status was assessed using the TCI [11]. The TCI divided the surface of the tongue into nine parts, and the adhesion status of the tongue coating was evaluated through visual inspection using a scoring system (0-2). A TCI of 50% or more (total score: ≥9) indicated poor oral hygiene.

Oral Moisture (OM)

Moisture on the dorsal tongue (10 mm from the tip) was measured three times using an oral moisture checker (Mucus, Life Co., Ltd., Osaka, Japan), and the average value was evaluated [12]. A value of <27.0 was considered indicative of oral dryness.

Maximum Occlusal Force (MOF)

The occlusal force was measured during a three-second maximum clench in the intercuspal position using a pressure-sensitive film (Dental Prescale II, GC Co., Tokyo, Japan) and an analysis device (Occluser, GC Co.) [13]. Denture wearers were assessed while wearing dentures. A MOF of <500 N indicated decreased occlusal force.

Tongue-Lip Motor Function (Oral Diadochokinesis {ODK})

The participants rapidly repeated the syllables “pa,” “ta,” and “ka” for five seconds. The number of repetitions per second was recorded using a counter (Kenkokun Handy, Takei Scientific Instruments Co., Ltd., Tokyo, Japan) [14]. A rate of <6.0 syllable/s for any syllable indicated reduced motor function.

Maximum Tongue Pressure (MTP)

The participants held the probe with a balloon lightly with the anterior teeth and pressed a balloon against the palate for seven seconds using a tongue pressure measurement device (TPM-01, JMS Co., Ltd., Hiroshima, Japan) [15]. The maximum value displayed was recorded. An MTP of <30 kPa was considered reduced.

Masticatory Function (MF)

Masticatory function was measured as the amount of glucose eluted during the chewing of a gummy jelly. The participants chewed a 2 g glucose-containing gummy jelly (Glucoram, GC Co.) for 20 seconds. The glucose concentration was measured using a chewing performance tester (Gluco Sensor GS-II, GC Co.) [16]. A value of <100 mg/dL indicated decreased masticatory function.

Swallowing Function (SF)

Swallowing function was assessed using the Eating Assessment Tool-10 (EAT-10) questionnaire (screening tool for dysphagia) [17]. EAT-10 consisted of 10 questions asked using a five-point scale (zero = no problem; four = severe problem). A total score of ≥3 indicated reduced swallowing function.

Statistical analysis

Participant characteristics were expressed as median (interquartile range) for continuous variables and as numbers (percentages) for categorical variables. For group comparisons, the age category (i.e., middle-aged, early older adult, and late older adult) and BMI group (nonunderweight versus underweight) were used as independent variables, and oral function assessments were used as dependent variables. The analysis was performed using a two-way ANOVA. The main and interaction effects were examined in the two-way ANOVA. The cell sizes were close to a symmetrical bell shape, and based on the central limit theorem and the robustness of ANOVA to mild non-normality, the application of parametric analysis was considered acceptable. Additionally, the participants with and without oral hypofunction were compared in terms of age, sex, medical history, the number of present teeth, FTUs, and each oral function. To account for potential confounding, additional analyses were performed using general linear models (GLM), with age category and BMI group as fixed factors, sex and medical history as additional fixed factors, and the number of present teeth as a covariate. Each oral function measure was entered separately as a dependent variable. When a significant interaction was detected, post hoc comparisons were conducted using the Bonferroni correction to examine simple effects within each BMI group. Statistical analyses were conducted using IBM SPSS Statistics version 30.0 (IBM Corp., Armonk, NY). A p-value of <0.05 was considered statistically significant.

## Results

Among the 113 patients initially recruited, six were excluded because of missing data, leaving 107 participants for analysis (43 men {40.2%} and 64 women {59.8%}) (Figure 1). The mean age of the cohort was 72.9 ± 9.2 years.

Table 1 shows the characteristics of the study participants. The mean BMI of the participants was 22.6 ± 4.1 kg/m². The main medical histories were coronary artery disease, hypertension, diabetes, and cancer. Among the 107 participants, 11 (10.3%) had no medical history, whereas 81 (75.7%) had at least one major systemic disease, such as cardiovascular disease, hypertension, diabetes, or cancer. Other chronic conditions (e.g., osteoarthritis and osteoporosis) were present in the remaining participants but were not classified under the “medical history” category in Table 1.

**Table 1:** Participant characteristics BMI, body mass index; CAD, coronary artery disease; HT, hypertension; DM, diabetes mellitus; nif-FTUs, functional tooth units composed of natural teeth, fixed prostheses, and implants

	Mean/n	SD/%
All participants	107	100.0
Age (years)	72.9	9.2
Sex		
Male	43	40.2
Female	64	59.8
BMI (kg/m²)	22.6	4.1
Medical History		
CAD	15	14.0
HT	30	28.0
DM	14	13.1
Cancer	22	20.6
Number of teeth	21.2	6.8
nif-FTUs	6.9	4.7

The prevalence of oral hypofunction in this population was 50.5% (Table 2). Among the participants categorized by the presence or absence of underweight, 48.1% of those in the nonunderweight group (n = 81) exhibited oral hypofunction. No significant differences were found in either mean BMI or low BMI (GLIM criteria for Asians) between the participants with and without oral hypofunction (Table 2).

**Table 2:** Oral function differences between those with and without oral hypofunction Continuous variables are expressed as mean ± SD and compared using Student’s t-test. Categorical variables are expressed as n (%) and compared using the chi-square test (p < 0.05). Low BMI was defined as <18.5 kg/m² for the participants aged <70 years and <20.0 kg/m² for those aged ≥70 years based on the GLIM criteria (for Asians) OM, oral moisture; TCI, tongue coating index; MOF, maximum occlusal force; MTP, maximum tongue pressure; ODK, oral diadochokinesis; MF, masticatory function; SF, swallowing function; BMI, body mass index; GLIM, Global Leadership Initiative on Malnutrition

	Nonoral hypofunction group, n = 53 (49.5%)		Oral hypofunction group, n = 54 (50.5%)		P-value
	Mean/n	SD/%	Mean/n	SD/%	
Age	70.3	10.1	75.0	7.4	0.003
Sex					
Male	21	39.6	22	40.7	0.906
Female	32	60.4	32	59.3	
BMI	22.9	2.3	22.3	3.8	0.435
Low BMI (GLIM criteria for Asians)	11	20.8	15	27.8	0.357
Oral hygiene-related items					
OM	29.3	1.5	28.3	2.4	0.006
TCI	24.4	22.5	41.2	26.1	<0.001
Oral function-related items					
MTP	33.8	7.4	26.3	6.3	<0.001
ODK, /pa/	6.5	0.8	5.7	1.0	<0.001
ODK, /ta/	6.5	0.7	5.8	0.8	<0.001
ODK, /ka/	6.0	0.9	5.3	0.8	<0.001
MOF	834.2	388.0	448.0	292.6	<0.001
MF	184.3	38.6	156.5	62.0	0.006
SF	0.7	1.3	3.2	5.3	0.002

Subjects with and without oral hypofunction showed significant differences across age and all examined oral function variables (p < 0.05; Table 2).

In these study subjects, only 26 (24.3%) were classified as underweight. When analyzed by age group, significant differences were observed in oral hygiene status (TCI) between the middle-aged and early older adult groups and between the middle-aged and late older adult groups. Significant differences in oral diadochokinesis (/ta/ and /ka/) were found between the middle-aged and late older adult groups. However, no significant differences in any oral function measures were observed between the underweight and nonunderweight groups (Table 3).

**Table 3:** Oral functions for each BMI status (nonunderweight and underweight) and each age group Classification of low BMI (underweight) based on the GLIM criteria. Middle-aged group (50-64 years, middle-aged group), early older adult group (65-74 years, y-old group), and late older adult group (≥75 years, o-old group). Two-way ANOVA (p < 0.05). All dependent variables are continuous ●Significant difference between comparison groups (p < 0.05) BMI, body mass index; nif-FTUs, functional tooth units composed of natural teeth, fixed prostheses, and implants; OM, oral moisture; TCI, tongue coating index; MOF, maximum occlusal force; MTP, maximum tongue pressure; ODK, oral diadochokinesis; MF, masticatory function; SF, swallowing function; GLIM, Global Leadership Initiative on Malnutrition

		Nonunderweight group			Underweight group			Main effects		Interaction	Post hoc (age)			
		N	Mean	SD	N	Mean	SD	BMI (GLIM)	Age	BMI (GLIM) × age	Middle-aged versus y-old	middle-aged versus o-old	Y-old versus o-old	
														Oral hygiene-related items														
Number of teeth	Middle-aged	15	21.9	6.9	4	23.8	5.7	0.499	0.744	0.963	-	-	-	
	Y-old	32	20.6	6.5	6	21.8	3.7	-	-	-	-	-	-	
	O-old	34	20.9	7.7	16	21.5	7.2	-	-	-	-	-	-	
	Total	81	21.0	7.0	26	21.9	6.2	-	-	-	-	-	-	
nif-FTUs	Middle-aged	15	7.7	5.0	4	7.5	5.7	0.928	0.856	0.911	-	-	-	
	Y-old	32	6.9	4.3	6	6.7	4.5	-	-	-	-	-	-	
	O-old	34	6.4	4.8	16	7.1	5.2	-	-	-	-	-	-	
	Total	81	6.8	4.6	26	7.1	4.9	-	-	-	-	-	-	
OM	Middle-aged	15	29.2	2.0	4	29.8	1.2	0.223	0.549	0.682	-	-	-	
	Y-old	32	28.8	2.0	6	29.0	1.5	-	-	-	-	-	-	
	O-old	34	28.2	2.3	16	29.3	1.8	-	-	-	-	-	-	
	Total	81	28.6	2.1	26	29.3	1.6	-	-	-	-	-	-	
TCI	Middle-aged	15	13.3	15.1	4	23.6	34.0	0.472	0.023	0.826	●	●	-	
	Y-old	32	33.0	25.9	6	32.4	23.1	-	-	-	-	-	-	
	O-old	34	38.2	26.1	16	42.3	24.4	-	-	-	-	-	-	
	Total	81	31.5	25.7	26	37.2	25.6	-	-	-	-	-	-	
Oral function-related items														
MTP	Middle-aged	15	35.0	9.8	4	30.3	8.7	0.282	0.07	0.694	-	-	-	
	Y-old	32	31.1	8.2	6	30.3	7.6	-	-	-	-	-	-	
	O-old	34	28.0	5.7	16	27.2	6.7	-	-	-	-	-	-	
	Total	81	30.5	7.9	26	28.4	7.1	-	-	-	-	-	-	
ODK, /pa/	Middle-aged	15	6.4	0.8	4	6.7	1.1	0.647	0.032	0.15	-	-	-	
	Y-old	32	6.1	1.1	6	6.6	0.5	-	-	-	-	-	-	
	O-old	34	6.1	0.9	16	5.6	0.9	-	-	-	-	-	-	
	Total	81	6.1	1.0	26	6.0	1.0	-	-	-	-	-	-	
ODK, /ta/	Middle-aged	15	6.5	0.8	4	7.0	1.0	0.259	0.015	0.763	-	●	-	
	Y-old	32	6.2	0.9	6	6.4	1.0	-	-	-	-	-	-	
	O-old	34	5.9	0.8	16	6.0	0.7	-	-	-	-	-	-	
	Total	81	6.1	0.9	26	6.2	0.9	-	-	-	-	-	-	
ODK, /ka/	Middle-aged	15	6.1	0.7	4	6.8	1.1	0.128	<0.001	0.588	-	●	-	
	Y-old	32	5.7	0.8	6	6.0	0.5	-	-	-	-	-	-	
	O-old	34	5.3	0.8	16	5.4	1.1	-	-	-	-	-	-	
	Total	81	5.6	0.9	26	5.8	1.1	-	-	-	-	-	-	
MOF	Middle-aged	15	553.6	343.0	4	862.3	528.1	0.543	0.783	0.224	-	-	-	
	Y-old	32	643.0	426.3	6	652.4	289.4	-	-	-	-	-	-	
	O-old	34	684.8	416.3	16	554.9	327.1	-	-	-	-	-	-	
	Total	81	644.0	405.9	26	624.7	356.0	-	-	-	-	-	-	
MF	Middle-aged	15	179.4	59.5	4	167.3	82.2	0.55	0.774	0.751	-	-	-	
	Y-old	32	160.1	56.4	6	164.5	30.4	-	-	-	-	-	-	
	O-old	34	180.7	47.7	16	163.2	54.5	-	-	-	-	-	-	
	Total	81	172.3	53.8	26	164.1	52.7	-	-	-	-	-	-	
SF	Middle-aged	15	0.4	0.9	4	0.8	1.0	0.714	0.34	0.941	-	-	-	
	Y-old	32	1.9	3.5	6	2.7	2.7	-	-	-	-	-	-	
	O-old	34	2.5	3.5	16	2.5	7.5	-	-	-	-	-	-	
	Total	81	1.9	3.3	26	2.3	6.0	-	-	-	-	-	-	

When these associations were further examined using multivariable-adjusted general linear models controlling for sex, medical history, and the number of present teeth, significant main effects of age group were observed for ODK (/ta/: p = 0.036; /ka/: p = 0.016). For TCI, no significant main effects of age group or underweight status were observed; however, a significant interaction between age group and underweight status was detected (p = 0.044). Post hoc analyses with the Bonferroni correction indicated that among nonunderweight participants, TCI differed significantly between middle-aged and early older adults and between middle-aged and late older adults (both p = 0.025), whereas no significant differences were observed among underweight participants.

## Discussion

This study demonstrated that nearly half of the patients had oral hypofunction even in the regular maintenance phase. Age and all oral function parameters had statistical significance between the oral hypofunction and normal function groups. Hence, specific characteristics such as equally low or high rates of oral hypofunction could not be revealed in this study population. Although no clear association was observed between oral hypofunction and underweight in this population, it was impossible to suggest oral hypofunction as a direct predictor of malnutrition among dental outpatients due to statistical weakness following the small underweight subgroup. Functional differences were observed only in tongue-related functions (TCI and ODK), even by age categories.

The prevalence of oral hypofunction in the regular dental maintenance phase is similar to previous reports (37.8% by Morinaga et al. [18] and 49.1% by Shirahase et al. [19]). Although the cause of declining oral function or how dental treatment restores their oral function is unknown because of the nature of the cross-sectional study, the 50.5% prevalence in a treated, regularly maintained cohort legitimately suggests that routine restorative/periodontal/prosthetic care is insufficient to keep all seven functions within normal ranges. In this study, 48.1% of the nonunderweight group also had oral hypofunction, and no significant difference in oral function level was observed between the nonunderweight and underweight groups. At least in this study population, (1) the oral hypofunction group is older than the nonoral hypofunction group, (2) there is a high prevalence of oral hypofunction even in the nonunderweight group, and (3) each oral function is not affected by age except tongue function. Although the incidence of oral hypofunction, as defined in the sum of hypofunction, is more observed with aging, each function is not suggested to decline with age. The study results were unable to conclude or suggest a relationship between oral hypofunction and malnutrition. As it might be considered that some factors, such as malnutrition, have a weak relationship with oral hypofunction or other factors, such as dietary habits, family support, and social participation, may compensate for their disadvantage of oral hypofunction, further longitudinal research is needed to clarify the mechanisms linking oral function and nutritional status [20,21].

The TCI and ODK showed significantly lower performance in the older groups than in the middle-aged group. TCI is considered to be influenced by reduced salivary secretion, decreased tongue mobility, papillary atrophy, and changes in oral hygiene behavior [22,23]. ODK reflects neuromuscular function, including tongue and lip motor speed, rhythm, and coordination [24]. ODK is affected not only by aging [24,25] but also by psychological factors [25] and cognitive function [25,26].

In the two-way ANOVA without covariate adjustment, age-related differences were observed primarily in TCI and ODK, whereas other oral functions did not show significant age-group differences. In the multivariable-adjusted analyses, age-related differences in ODK persisted, suggesting a relatively independent association with age. In contrast, TCI did not show a uniform age-group difference after adjustment; however, a significant interaction between age group and underweight status was observed. Specifically, age-related differences in TCI were evident only among nonunderweight participants, suggesting that nutritional status may modify the association between age and tongue coating. These findings indicate that age-related changes in oral hygiene indicators may not be uniform across nutritional strata.

The limitations of this study are as follows. First, this was a cross-sectional study, and longitudinal factors such as aging, the period of illness, or the term of recovery could not be considered. Second, although this study was conducted at the dental section in a university hospital that mainly accepted not serious or complicated patients, similar to those seen in general dental clinics, the generalizability of the findings may be limited. Third, swallowing function was assessed using the EAT-10 questionnaire, which is a subjective screening tool, so no objective swallowing assessment was performed. However, as it is an admitted evaluation method for the determination of oral hypofunction, the impact was considered to have been limited to evaluating oral hypofunction [1]. Fourth, initial oral status, the type of dental treatment that has been conducted, dietary intake, and known risk factors for malnutrition, such as education level, living alone, marital status, income, and occupation, were not evaluated in detail [27-30]. As the covariates included in this study may not be adequately considered, further study that includes these factors is required. Fifth, although the use of BMI as an indicator of nutritional status is simple to apply in a dental clinical setting, it does not reflect the multifactorial aspects of malnutrition, including those captured by other nutritional indicators, such as muscle mass, dietary intake, and inflammatory status [18]. Finally, the total sample size was relatively small, and a smaller number of underweight participants than expected were observed. A post hoc power analysis revealed that given the fixed sample size (N = 107), the statistical power to detect the association between oral function and underweight was approximately 0.62. Therefore, the study may have been underpowered to detect small-to-moderate effects, and the possibility of a type II error cannot be excluded.

## Conclusions

Approximately half of the dental outpatients in the maintenance phase in this study exhibited oral hypofunction. Oral hypofunction was significantly observed in elderly people in the dental maintenance phase. We did not detect a statistically significant uniform association between oral hypofunction and underweight, but the small underweight group limits our ability to rule out a clinically meaningful relationship. Further longitudinal research is needed to clarify the mechanisms linking oral function and nutritional status.
